# Clinician-researcher’s perspectives on clinical research during the COVID19 pandemic

**DOI:** 10.1371/journal.pone.0243525

**Published:** 2020-12-09

**Authors:** Sarah L. Silverberg, Lisa M. Puchalski Ritchie, Nina Gobat, Alistair Nichol, Srinivas Murthy

**Affiliations:** 1 Department of Pediatrics, University of British Columbia, Vancouver, British Columbia, Canada; 2 Department of Medicine, University of Toronto, Toronto, Ontario, Canada; 3 Li Ka Shing Knowledge Institute, St. Michael’s Hospital, Toronto, Ontario, Canada; 4 Department of Emergency Medicine, University Health Network, Toronto, Ontario, Canada; 5 Institute of Health Policy, Management and Evaluation, University of Toronto, Toronto, Ontario, Canada; 6 Nuffield Department of Primary Care Health Sciences, University of Oxford, Oxford, United Kingdom; 7 Clinical Research Centre, St. Vincent’s University Hospital, University College Dublin, Dublin, Ireland; 8 Australian and New Zealand Intensive Care Research Centre, Monash University, Melbourne, Australia; 9 The Alfred Hospital Intensive Care Unit, Melbourne, Australia; Xiangya Hospital Central South University, CHINA

## Abstract

**Objectives:**

The outcome of well-performed clinical research is essential for evidence-based patient management during pandemics. However, conducting clinical research amidst a pandemic requires researchers to balance clinical and research demands. We seek to understand the values, experiences, and beliefs of physicians working at the onset of the COVID-19 pandemic in order to inform clinical research planning. We aim to understand whether pandemic settings affect physician comfort with research practices, and how physician experiences shape their understanding of research in a pandemic setting.

**Methods:**

A survey tool was adapted to evaluate familiarity and comfort with research during a pandemic. A cross-sectional, online questionnaire was distributed across Canadian research networks early in the COVID-19 outbreak. The survey was administered between March 11^th^ and 17^th^, 2020, during a time of local transmission but prior to the surge of cases. We aimed to recruit into the survey physicians in infectious disease and critical care research networks across Canada.

**Results:**

Of the 133 physician respondents, 131 (98%) considered it important to conduct clinical research during the COVID-19 pandemic. Respondents were more accepting of adaptations to the research process in during a pandemic compared to in a non-pandemic setting, including conducting research with deferred consent (χ^2^ = 8.941, 95% CI: -0.264, -0.085, p = 0.003), using non-identifiable observational data with a waiver of consent with a median score of 97 out of 100 (IQR: 79.25–100) vs median 87 out of 100 (IQR: 63–79) (95% CI: -12.43, 0.054, p = 0.052). The majority felt that research quality is not compromised during pandemics.

**Conclusions:**

Physicians consider it important to conduct research during a pandemic, highlighting the need to expedite research activities in pandemic settings. Respondents were more accepting of adaptations to the research process for research conducted during a pandemic, compared to that conducted in its absence of a pandemic.

## Introduction

One challenge posed by the recent SARS-nCOV-2 (COVID-19) outbreak is how to effectively and efficiently conduct research to better improve clinical care of infected patients.

Conducting research during a pandemic is difficult, and associated with a number of pitfalls, including busy staff, scared patients, and concerns for infection control [1]. Yet it is essential to an effective pandemic response, as evidenced from the number of trials ongoing [2]. The time frame for generating knowledge is compressed and urgent due to the novelty of the disease, and physicians must often care for patients with little or no evidence to guide clinical decisions [3]. Historically, there has been insufficient capability to generate evidence on a timely basis during pandemics, resulting in gaps in pandemic preparedness and response [4]. With each outbreak, the global community gains new skills and strategies for rapidly executing scientific research and identifies new gaps in evidence-gathering responses [5,6]. If instituted early in a pandemic’s course, novel research has the potential to dramatically change the trajectory of the outbreak [7–9].

At the same time, there is public demonstration of support in participating in randomized trials during a pandemic, perceiving that the benefit obtained outweighs the risks [4]. Balancing these challenges, given the number of competing interests, has proven difficult, and this current outbreak poses a unique opportunity to better understand these issues, as well as develop approaches to overcome them.

Previous studies, particularly around the H1N1 outbreak, have focused on healthcare worker perceptions and attitudes towards pandemic response measures, as well as perceptions of both public and healthcare providers towards vaccinations [10–12]. Few studies have examined the perspectives on, and barriers to, conducting research in a pandemic setting [7]. Clinicians, in particular, create a critical team, providing both front-line care as well as participation in clinical research activities. They must balance the clinical burden of their department, the needs of their patients, and the needs of a potential trial, amongst other factors. It is therefore essential to understand their experiences and beliefs regarding conducting research in the midst of a pandemic. In particular, trials regarding therapies for patients critically ill with COVID-19, where the need to make decisions regarding novel therapies is most acute, directly impact the care provided by critical care and infectious disease physicians. In this study, we sought to better understand the experiences and beliefs of Canadian physicians who are likely to be simultaneously be providing care for COVID-19 patients as well as enrolling them in clinical trials in the difficult setting of a pandemic. Our survey focuses on questions in two domains: 1) experiences conducting research in critical care and outbreak scenarios, and 2) beliefs regarding research in pandemic and non-pandemic settings.

## Materials and methods

We conducted a cross-sectional, online survey to rapidly assess the views of Canadian physician researchers who were actively working on COVID-19 preparedness planning (S1 Appendix). We adapted a survey used in 2013, in the absence of a pandemic, across European countries, to assess comfort with enrolling patients in research studies during an outbreak. The survey has been validated and used across a number of regions previously.

The survey was rapidly adapted for the Canadian and current COVID-19 context. Optimization occurred via clinical sensibility testing and assessing for face and content validity by lay-people and experts [13]. The survey was validated by test-retest validation by colleagues and piloted for face validity. The survey comprised of collection of basic respondent characteristics without collecting identifying data, as well as a series of closed-ended questions on 3-point and 5-point Likert scales, as well as scales from 0–100 eliciting the beliefs and perspectives regarding research in pandemic and non-pandemic settings. In particular, we focused on comparing beliefs regarding three research practices in pandemic vs. non-pandemic settings: 1) comfort randomizing patients using deferred consent for medications not proven to be safe in the patient population, 2) belief in collecting non-identifiable observational patient data to inform clinical management using a waiver of consent, and 3) comfort enrolling patients into a study using current treatments, with follow-up using routinely collected data under a waiver of consent. All questions regarding research in pandemic settings were not specific to the SARS-nCOV-2 pandemic.

We distributed this survey by email and social media channels across primarily acute and critical care research networks, across jurisdictions, with a focus on dissemination to Canadian infectious disease and critical care physicians, via a convenience sample via snowball sampling of clinicians and investigators. We distributed the survey via a Canadian infectious disease listserv as well as slack channel, as well as via a Canadian ICU clinician listserv and slack channel. Our survey is representative of physicians working in acute and critical care due to the nature of sampling. Due to the nature of the type of COVID-19 research being conducted in Canada at the time of the survey, we focused on physicians treating patients hospitalized or presenting to hospital with COVID-19. No restrictions on the type of patients being cared for were set. We focused on infectious diseases, general internal medicine, or intensive care physicians’ in academic hospitals in Canada, who from our experience represent the primary physicians caring for these patients in hospital and would be most likely to be involved in hospital-based research. We did not restrict our sample to solely clinician-investigators or researchers in order to capture the perspectives of a diverse pool of individuals potentially involved in the implementation of research into practice via a non-biased sample. No specific inclusion or exclusion criteria were set; however, due to the nature of survey distribution, the survey only included physicians belonging to acute and critical care research networks in Canada.

The survey was rapidly distributed between March 11th and 17th, 2020, at the onset of COVID-19’s emergence in Canada. The survey was disseminated through email and Slack across infectious diseases and critical care discussion groups amongst Canadian physicians over this time-period. These networks represent trialists as well as clinicians, and reflect some of the diverse group of individuals who are typically involved in clinical research. Study data were collected and managed using REDCap electronic data capture tools at BC Children’s Hospital [14]. At the time of data collection, Canada was experiencing local transmission of COVID-19, but no large surge in hospitalizations [15]. All questionnaires were self-administered digitally, with voluntary participation. The protocol was approved by the Research Ethics Board of BC Children’s Hospital (Vancouver, Canada) (Reference number: H20-00438), with implied consent with participation. We followed the Checklist for Reporting Results of Internet E-Surveys (CHERRIES Checklist) (S2 Appendix).

### Statistical analysis

Our analysis is primarily descriptive, according to the questions asked in the survey. Incomplete surveys were included for the questions to which they contributed. We present descriptive statistics as proportions as well as means (+/- SD) for continuous variables or medians (IQR) for discrete scales. Three-point and five-point Likert scales were used to assess agreement with statements, with three indicating ‘a large effect,’ and five indicating ‘strongly agree,’ ‘very comfortable,’ or ‘very important.’ A sliding scale (0–100) was used to further elucidate the strength of conviction for certain statements. Our analysis focused primarily on comparing perceptions on research performed during an outbreak, compared with that performed in the absence of an outbreak.

We compared categorical responses using chi-square tests (alternatively, Fisher exact tests for expected values <5) and Kruskal-Wallis tests, and continuous measures using the Student t test and Mann-Whitney U test depending on data distribution. We hypothesized that there should be no difference in comfort with research practices between pandemic and non-pandemic settings, experience recruiting, or by clinical specialty. For comparison of multiple continuous variables, we used a simple linear regression, while one-way ANOVA was used when comparing continuous ordinal responses.

## Results

### Respondent characteristics

We analysed responses from 145 physicians collected in March 2020, with a 92% participation rate (133 physicians answered at least one question of the survey) and 71% survey completion rate. Respondents were infectious diseases and critical care clinicians on email and Slack discussion groups in February and March 2020. Response rate was incalculable due to the nature of social network survey distribution. Respondents clinically worked in critical care (n = 32), infectious diseases (n = 76), general pediatrics and general internal medicine (n = 11), and a mixture of other fields (n = 14) (Table 1). On average, respondents were more likely to consider themselves more strongly clinicians rather than researchers (on scale of 0–100, where 0 represents clinician and 100 represents researcher: median 25, inter-quartile range (IQR) 25–50). The majority (69.9%) of respondents had experience recruiting patients into research studies, with a median comfort level of 4 (IQR = 2–5) recruiting hospitalized patients into randomized trials on a 5-point Likert scale. There was strong agreement that it is important to recruit hospitalized patients into randomized trials in a pandemic setting, with only 1.5% (n = 2) respondents not agreeing that it was somewhat or very important (Table 1).

**Table 1:** Respondent experience and baseline perspectives on research during pandemics.

**RESPONDENT CHARACTERISTICS AND EXPEIRENCES**	
**Primary clinical specialty (n = 133), N (%)**	
Critical care	32 (24.1)
Infectious diseases	76 (57.1)
General internal medicine and general pediatrics	11 (8.27)
None of the above	14 (10.53)
**Consideration of self as clinician (score of 0) vs researcher (score of 100) (n = 103)**	
Median (IQR)	25 (25–50)
Mean (SD)	30.75 (25.86)
**Experience recruiting patients into research studies (n = 133), N (%)**	
Yes	93 (69.9)
No	40 (30)
**Comfort recruiting hospitalized patients into randomized trials (n = 132), N (%)**	
Very uncomfortable	18 (13.6)
Somewhat uncomfortable	24 (18.2)
Neutral	23 (17.4)
Somewhat comfortable	30 (22.7)
Very comfortable	37 (28.0)
**Involved in care of critically ill patients during previous outbreaks (n = 133), N (%)**	
Yes	60 (45)
No	73 (54.8)
**There has been a patient with suspected or confirmed COVID19 admitted to your hospital (n = 133), N (%)**	
Yes	66 (49.6)
No	67 (50.4)
**Cared for patient with suspected or confirmed COVID19 (n = 133), N (%)**	
Yes	30 (22.5)
No	103 (77.4)
**RESPONDENT PERSPECTIVES AND BELIEFS**	
**How important is it to recruit hospitalized patients into randomized trials during a pandemic? (n = 133), N (%)**	
Not important at all	2 (1.5)
Somewhat unimportant	0
Neutral	0
Somewhat important	27 (20.3)
Very important	104 (78.2)
**Comfort randomizing hospitalized COVID-19 patients to a no-treatment arm (control arm) with a study looking at experimental antiviral therapy (n = 108), N (%)**	
Very uncomfortable	6 (5.6)
Somewhat uncomfortable	17 (15.7)
Neutral	16 (14.8)
Somewhat comfortable	30 (27.78)
Very comfortable	39 (36.1)
**How large a factor does the increased clinical workload during a major outbreak impact ability to recruit patients into a clinical trial (n = 107), N (%)**	
No effect at all	1 (0.1)
A small effect	64 (59.8)
A large effect	42 (39.3)
**To what extent would recruiting patients to a pandemic-specific clinical trial threaten current ongoing research activities in your unit? (n = 107), N (%)**	
No effect at all	41 (38.3)
A small effect	51 (47.7)
A large effect	15 (14.0)
**To what extent would recruiting patients to a pandemic-specific clinical trial threaten current clinical care in your unit? (n = 108), N (%)**	
No effect at all	30 (27.8)
A small effect	71 (65.7)
A large effect	7 (6.5)
**Because of the difficulty in enrolling and monitoring patients, research conducted during pandemics is of lower quality than that conducted in the absence of a pandemic (n = 133), N (%)**	
Strongly disagree	32 (24.06)
Somewhat disagree	76 (57.14)
Neutral	11 (8.27)
Somewhat agree	14 (10.53)
Strongly agree	0
**In the** **absence of a pandemic****, do you believe that non-identifiable observational data from patients to inform clinical management should be collected with a waiver of consent? (n = 103)**	
Median (IQR)	87 (63–79)
Mean (SD)	79.07 (23.01)
**During a pandemic,** **do you believe that non-identifiable observational data from patients to inform clinical management should be collected with a waiver of consent? (n = 102)**	
Median (IQR)	97 (79.25–100)
Mean (SD)	85.25 (22.31)
**In the** **absence of a pandemic****, do you feel comfortable randomizing your patients using deferred consent for an antiviral medication that has not been proven to be safe in your patient population? (n = 107), N (%)**	
Yes	30 (28.0)
No	77 (71.9)
**During a pandemic****, do you feel comfortable randomizing your patients using deferred consent for an antiviral medication that has not been proven to be safe in your patient population? (n = 107), N (%)**	
Yes	63 (58.9)
No	44 (41.1)
**How would you feel about enrolling patients in an ethically approved study where patients are randomised to 1 of 2 currently used and acceptable treatments (e.g. steroid administration) and then followed up using routinely collected data, under a waiver of consent, in the** **absence of a pandemic****? (n = 106), N (%)**	
Very uncomfortable	13 (12.3)
Somewhat uncomfortable	19 (17.9)
Neutral	10 (9.4)
Somewhat comfortable	30 (28.3)
Very comfortable	34 (32.1)
**How would you feel about enrolling patients in an ethically approved study where patients are randomised to 1 of 2 currently used and acceptable treatments (e.g. steroid administration) and then followed up using routinely collected data, under a waiver of consent,** **during a pandemic****? (n = 107), N (%)**	
Very uncomfortable	5 (4.7)
Somewhat uncomfortable	14 (13.1)
Neutral	12 (11.2)
Somewhat comfortable	34 (31.8)
Very comfortable	42 (39.3)

While just under half (n = 60) of respondents had been involved in caring for critically ill patients during a prior outbreak, only 22.5% (n = 30) had thus far cared for a suspected or confirmed case of COVID-19 at time of completion, although half (n = 66) noted that such a case did exist thus far in their hospital.

### Interaction between research and clinical work

Regarding the threat towards ongoing research activities, 14% of respondents (n = 15) indicated that pandemic research would have a large effect, but 38.3% (n = 41) felt it would have no effect at all. With respect to clinical care, 65.7% (n = 71) believed it would have a small effect on clinical care, and only 6.5% (n = 7) believed it would have a large effect. The majority of respondents (59.8%, n = 64) felt that the increased clinical workload would have a small effect on their ability to recruit patients into clinical studies. Respondents indicated that conducting pandemic-specific trials in clinical settings will impact other non-pandemic related ongoing research significantly more than it will impact clinical duties in their own clinical environments, rather than affecting them similarly (Mann-Whitney U test = 27874, p = <0.001).

### Perspectives on conducting research in a pandemic setting

**Fig 1:**
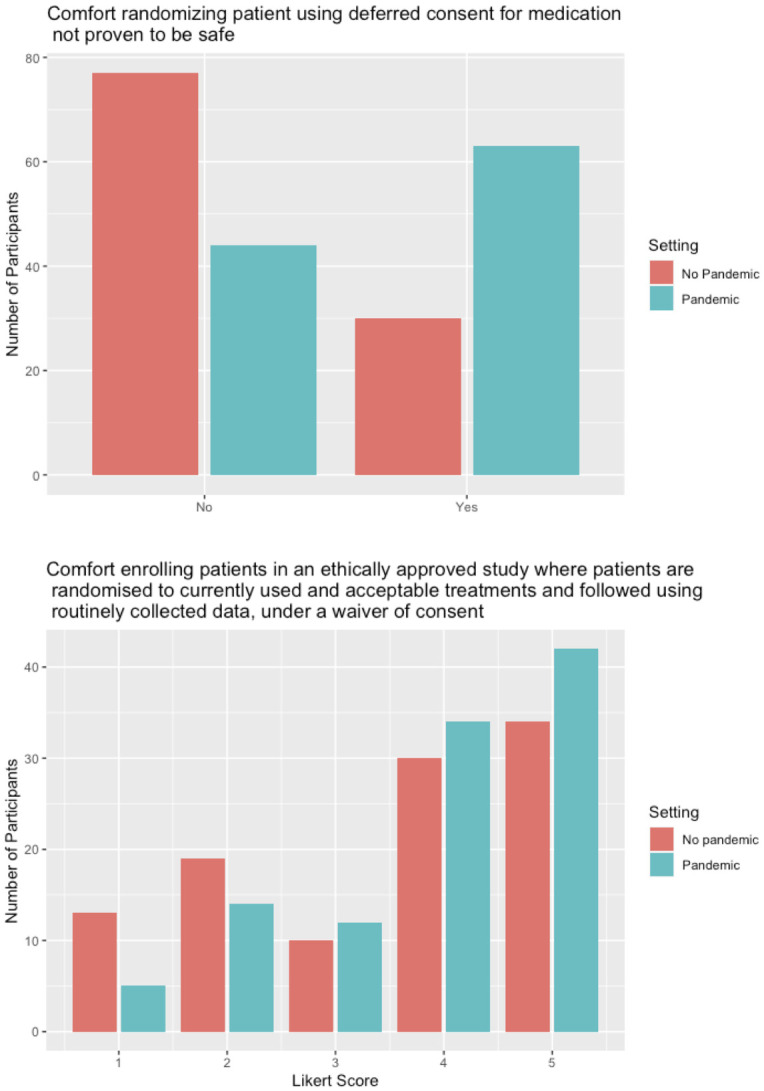
Comparison of comfort with research practices in pandemic and non-pandemic settings. (A) Comfort randomizing patients using deferred consent for an unproven medication, in which participants were more comfortable in pandemic settings than non-pandemic settings. (B) Comfort randomizing patients to a current therapy with routine data collection follow up, under a waiver of consent, which did not differ amongst participants between pandemic and non-pandemic settings.

In comparisons of comfort with various models of consent for research practices, physicians were more comfortable with the practice in the setting of a pandemic than they were in non-pandemic settings. When randomizing patients using deferred consent for an antiviral medication that has not been proven to be safe in the patient population, only 28% (n = 30) were comfortable doing so in a non-pandemic setting, while 58.9% (n = 63) were comfortable doing so during a pandemic (χ^2^ = 8.941, 95% CI: -0.264, -0.085) (p = 0.003) (Fig 1A). When asked how strongly they believe on a scale from 0–100 that non-identifiable observational data from patients to inform clinical management should be collected with a waiver of consent, responding with a median score of 97 (IQR: 79.25–100) during a pandemic and a median score of 87 (IQR: 63–79) in the absence of a pandemic. Respondents were more comfortable with this practice in the presence of a pandemic (T-statistic = -1.955, 95% CI: -12.43, 0.054) (p = 0.052). Respondents comfort with this practice during a pandemic varied depending on whether respondents had experience recruiting patients into studies (T-statistic = -2.20, 95% CI: -25.67, -0.97, p = 0.035) (Table 2). When asked about enrolling patients in an ethically approved study where patients are randomised to 1 of 2 currently used and acceptable treatments and then followed up using routinely collected data, under a waiver of consent, respondents were not significantly more comfortable with doing so during a pandemic (median of 4 out of 5, IQR: 2–5) than not (median of 4 out of 5, IQR: 3–5) (Mann-Whitney U test = 4960, p = 0.079) (Fig 1B).

**Table 2:** Whether experience recruiting patients into studies affects your beliefs on research when there is a pandemic.

		Experience recruiting patients into studies before	p
Do you feel comfortable randomizing patients using deferred consent for an antiviral medication that has not been proven to be safe in the patient population?^1^	No pandemic	χ^2^ = 0.000	p = 1.0
		95%CI:-0.21, 0.19	
	Pandemic	χ^2^ = 0.81	p = 0.368
		95%CI: 0.32, 0.44	
Do you believe that non-identifiable observational data from patients to inform clinical management should be collected with a waiver of consent?^2^	No pandemic	T = -1.56	p = 0.128
		95%CI: -20.33, 2.66	
	Pandemic	T = -2.20	p = 0.035
		95%CI: -25.67, -0.97	
How would you feel about enrolling patients in a study where patients are randomised to 1 of 2 currently used and acceptable treatments, and then follow up using routinely collected data under a waiver of consent?^3^	No pandemic	U = 994	p = 0.592
	Pandemic	U = 1073	p = 0.809

^1^Results represent the outcome of a chi squared test.

^2^Results represent the student t-statistic.

^3^Results represent the Mann-Whitney U Statistic.

Almost 64% (n = 69) felt somewhat or very comfortable randomizing hospitalized COVID-19 patients into a no-treatment arm of a study looking at experimental antiviral therapy. Respondents disagreed that research conducted during pandemics is of lower quality, with 81.2% (n = 108) disagreeing or strongly disagreeing that it is of lower quality.

### Clinician vs. researcher perspectives on research during outbreaks

There were few differences between respondents who self-identified more as a clinician than as a researcher. Of note, those who identified more strongly as researchers were more comfortable with the statement that observational data from patients should be collected with a waiver of consent to inform clinical management in both the absence and presence of a pandemic (β = 0.221, p = 0.0125; and β = 0.237, p = 0.007 respectively).

### Experience and effects on beliefs

There were few differences in beliefs amongst those with experience recruiting patients into studies or caring for critically ill patients during previous outbreaks. Those with previous experience recruiting patients believed more strongly that observational data from patients in a pandemic should be collected with a waiver of consent (T-statistic = -2.20, 95% CI: -25.67, -0.97, P = 0.035) (Table 2), while other past experiences did not correlate with the strength of this belief or other beliefs. There was generally no difference in opinion on using these research methods in pandemics vs non-pandemic settings amongst those who had cared for COVID-19 patients or whose hospitals had patients admitted with COVID-19. However, those who work in hospitals that have had patients admitted with COVID-19 felt more strongly that research conducted in a pandemic setting is of lower quality (Mann-Whitney U test = 1020, p = 0.007).

### Respondent perspectives by specialty

There were some differences in comfort and beliefs around research by specialty. Critical care physicians believed less strongly than infectious disease physicians that observational data from patients should be collected with a waiver of consent, in both pandemic and non-pandemic scenarios (F(3,98) = 3.375, p = 0.022 and F(3,99) = 3.176 p = 0.028 respectively) (Table 3). There was no difference in respondents’ comfort randomizing patients using deferred consent. While there was an overall difference in opinion regarding enrolling patients in an ethically approved study where patients are randomised to 1 of 2 currently used and accepted treatments and then followed up using routinely collected data, under a waiver of consent in both non-pandemic and pandemic settings (χ^2^ = 9.635, p = 0.022 and, χ^2^ = 8.235, p = 0.041 respectively), no specific between-group differences account for these differences (Table 3). However, critical care physicians felt more strongly that recruiting patients to pandemic-specific trials would threaten other ongoing clinical care, much more so than other physician groups (χ^2^ = 12.82, p = 0.005). There were no significant differences in comfort randomizing COVID-19 patients to experimental controls, and no differences in perspectives on how clinical workloads during pandemics might affect ability to recruit patients into trials (or vice versa). There was no difference by specialty regarding whether research in a pandemic is of lesser quality.

**Table 3:** Perspectives on research in pandemic settings by specialty.

		Primary Clinical Specialty^1^	P
Do you feel comfortable randomizing patients using deferred consent for an antiviral medication that has not been proven to be safe in the patient population?^2^	No pandemic	χ^2^ = 0.005	p = 0.941
	Pandemic	χ^2^ = 0.341	p = 0.559
Do you believe that non-identifiable observational data from patients to inform clinical management should be collected with a waiver of consent?^3^	No pandemic	F(3,99) = 3.176	p = 0.028
	Pandemic	F(3,98) = 3.375	p = 0.022
How would you feel about enrolling patients in a study where patients are randomised to 1 of 2 currently used and acceptable treatments, and then follow up using routinely collected data under a waiver of consent?^4^	No pandemic	χ^2^ = 9.635	p = 0.022
	Pandemic	χ^2^ = 8.235	p = 0.041
For hospitalized patients with COVID-19 infection, how comfortable do you feel randomizing patients to a no-treatment arm (control arm) with a study looking at experimental antiviral therapy?^4^		χ^2^ = 1.100	p = 0.777
How large a factor does the increased clinical workload during a major outbreak impact your ability to recruit patients into a clinical trial?^4^		χ^2^ = 2.515	p = 0.473
To what extent would recruiting patients to a pandemic-specific clinical trial threaten current ongoing research activities in your unit?^4^		χ^2^ = 12.82	p = 0.005
To what extent would recruiting patients to a pandemic-specific clinical trial threaten current clinical care in your unit?^4^		χ^2^ = 3.43	p = 0.330
Because of the difficulty in enrolling and monitoring patients, research conducted during pandemics is of lower quality than that conducted in the absence of a pandemic.^4^		χ^2^ = 3.63	p = 0.459

^1^Primary clinical specialty divided into a) critical care, b) infectious diseases, c) general internal medicine and general paediatrics, and d) other.

^2^Results represent the outcome of a chi squared test.

^3^Results represent the outcome of an ANOVA.

^4^Results represent the outcome of a Kruskal-wallis chi squared test.

## Discussion

Our survey of physicians who are involved in clinical care and research on patients affected by outbreaks highlights important insights into the perspectives of conducting research in challenging times. Overall, respondents consider it important to actively participate in randomized control trials as a means to gather evidence during a pandemic. In our study, more than two thirds of respondents noted that the pandemic would affect clinical care and workload speaks to the need for health care worker pandemic preparedness at the clinical unit and hospital level. Our survey evaluated the potential impact of COVID-19 on other research and clinical duties, finding that just under two thirds of respondents felt as though it would have some effect on ongoing research activities. This sentiment was particularly strong amongst critical care physicians.

Our study demonstrates a difference regarding conducting research in pandemic settings compared to non-pandemic settings. In two out of three scenarios, respondents felt much more comfortable in the setting of a pandemic using deferred consent for experimental medications, and collecting non-identifiable observational data without consent. As well, our survey demonstrated concern amongst respondents that research conducted in pandemic settings is of lower quality; with the large amount of data emerging on COVID-19, this concern may be valid. Of note, this survey was taken in the weeks leading up to large patient surges in the targeted geographic region.

Our findings, that almost two thirds of respondents felt as though COVID-19 impacts ongoing research activities, contrasts to findings from previous studies conducted during the H1N1 outbreak, which indicated that only one third of evaluated research efforts slowed [7]. Perhaps higher number of expected ICU admissions during COVID-19, compared to the milder H1N1 influenza, account for such a difference; no similar study was performed in Canada during the SARS outbreak in 2003. As well, the completion of our survey during the emergence of the COVID-19 pandemic in Canada, rather than later on in the pandemic’s course, may account for the difference.

Our findings echo previous work emphasizing the need for research protocols to be present to allow for effective research during pandemics [16,17]. In the midst of a pandemic, careful consideration should be taken for assessment of potentially compromised care and opportunity to mitigate the effects of the pandemic on other aspects of clinical care. Previous studies have highlighted how ongoing work between pandemics by research consortiums will allow for more streamlined initiation of research in pandemic settings; this may potentially reduce the impact of pandemic-related work on other duties [18].

The ethical standards of research conducted during an outbreak, as stated by the World Health Organization, should be maintained, and the urgency or a public health emergency does not justify lower ethical standards [19,20]. Our survey has demonstrated that the pandemic context does influence what is perceived as ethically acceptable practice and can shift perspectives on what is tolerable risk vs. benefit when conducting research in urgent settings. The potential illness severity, as well as elements of disease novelty and unclear disease course faced by patients with COVID-19 may have influenced the liberalization of comfort with research practices during the pandemic [21]. The sense of urgency for producing results, as well as the potential to influence the care of many patients, can change the perception of ethical standards. Despite desires to provide possible treatments to patients who may be critically unwell, maintaining standards for ethical research, including consent processes, remains vital [22,23].

No national guidelines were woven into this survey beyond the standards of human research ethics. As our understanding of the management of COVID-19 develops further, physician perspectives regarding acceptable treatment options is likely to change.

Our survey has several strengths. We used pragmatic methods to receive feedback from a sample of respondents likely to be providing clinical care to COVID-19 patients as well as conduct research with this population. We modified a survey tool previously used in other regions, allowing us to accurately compare perspectives in our cohort. As it was conducted relatively early in the COVID-19 outbreak, prior to many physicians directly caring for COVID-19 positive patients and being faced with these potentially difficult scenarios, this survey allowed us to identify their thoughts, feelings and beliefs without them being clouded by their recent and direct actions. However, with the COVID-19 crisis imminently looming, it provided the necessary context for these questions to be asked beyond a hypothetical. Notwithstanding, our survey also had limitations. First, by not collecting demographic data we are not able to accurately assess how representative the population of respondents is compared to care providers, including by country of origin. However, given the nature of distribution of this study through specific physician networks, our findings are likely themselves more reflective of these networks themselves. Indeed, similar networks have been cited as key for conducting research in pandemic settings [24,25]. Their generalizability to broader, international physician cohorts is unknown; research preferences vary by region and ensuring ethics boards and policymakers understand local preferences is crucial. Due to the needed expediency of conducting this survey prior to the peak of the COVID-19 outbreak, these networks were critical to evaluating the beliefs of those most likely to be making these decisions in real-time. Finally, our findings might reflect stated, rather than actual experiences of respondents, which is a recognized limitation of survey methodology [26].

Our study demonstrates the need for ongoing work to better understand the perspectives and preferences of patients and the public, who are integral to ongoing research practices, in research participation during pandemics. As well, while our survey represents the views of clinicians and researchers at the outset of a pandemic, ongoing evaluation of the experiences of clinicians and researchers recruiting for clinical trials during COVID-19 will be informative. This includes the need to better understand how clinicians and researchers balance the demands of clinical care and research during these times. Finally, repeating surveys like ours later on in the course of a pandemic, particularly after more respondents have actively cared for patients affected by the illness, may allow for an opportunity to explore shifting perspectives as a result of experience.

## Conclusions

Amongst a network of physicians likely to be involved in the clinical care and/or research related to COVID-19 patients, there are differences in comfort level conducting research in pandemic situations compared to non-pandemic situations. Conducting high-quality research during a pandemic is perceived as a major priority, and education regarding the challenges of outbreak-specific research is important to better serve the clinical and research community and inform study design.

## Supporting information

S1 Appendix COVID-19 researcher survey. (PDF)

S2 Appendix CHERRIES checklist. (DOCX)
