# Supplementary material for: Clinician-researcher’s perspectives on clinical research during the COVID19 pandemic
Source: PLoS One. 2020 Dec 9;15(12):e0243525. doi: 10.1371/journal.pone.0243525 (PMC7725301; doi:10.1371/journal.pone.0243525)
Supplement: S1 Appendix — (PDF) [file pone.0243525.s001.pdf]

COVID 19 Researcher

Thank you for participating in this survey to better understand how we perform clinical research during a pandemic. Given the ongoing Covid-19 outbreak, it is timely that we generate data that reflects current perspectives on research during a major respiratory infection outbreak.

The survey should take around five to ten minutes to complete. All of your answers will be anonymous, and no identifiable information is being collected. By completing the survey, you imply that you consent to this project. All data will be securely stored, and then destroyed one year after it is analyzed.

This survey has been approved by the ethics board at the University of British Columbia, Canada. If you have any concerns or complaints about your rights as a research participant, please call the Office of Research Ethics at 604-822-8598 (Toll Free: 1-877-822-8598 or email RSIL@ors.ubc.ca), and mention study number H20-00438

1)	1. Have you recruited patients into research studies before?	◯ Yes<br>◯ No
2)	How comfortable are you with recruiting hospitalized into randomized controlled trials?	◯ Very uncomfortable<br>◯ Somewhat uncomfortable<br>◯ Neutral<br>◯ Somewhat comfortable<br>◯ Very comfortable
3)	How important is it to recruit hospitalized patients into randomized trials during a pandemic?	◯ Not important at all<br>◯ Somewhat unimportant<br>◯ Neutral<br>◯ Somewhat important<br>◯ Very important
4)	Have you been involved with the care of critically ill patients during the H1N1, MERS-CoV or SARS outbreaks previously?	◯ Yes<br>◯ No
5)	Has a patient with suspected or confirmed COVID-19 been admitted to the hospital in which you work?	◯ Yes<br>◯ No
6)	Have you cared for a patient with suspected or confirmed COVID-19 infection?	◯ Yes<br>◯ No
7)	What is your primary clinical specialty?	◯ Critical Care<br>◯ Infectious Diseases<br>◯ General Pediatrics<br>◯ General Internal Medicine<br>◯ Emergency Medicine<br>◯ None of the above

Research during pandemics

8) For hospitalized patients with COVID-19 infection, how comfortable do you feel randomizing patients to a no-treatment arm (control arm) with a study looking at experimental antiviral therapy?

- ◯ Very uncomfortable
- ◯ Somewhat uncomfortable
- ◯ Neutral
- ◯ Somewhat comfortable
- ◯ Very comfortable

9) How large a factor does the increased clinical workload during a major outbreak impact your ability to recruit patients into a clinical trial?

- ◯ No effect at all
- ◯ A small effect
- ◯ A large effect

10) To what extent would recruiting patients to a pandemic-specific clinical trial threaten current ongoing research activities in your unit?

- ◯ No effect at all
- ◯ A small effect
- ◯ A large effect

11) To what extent would recruiting patients to a pandemic-specific clinical trial threaten current clinical care in your unit?

- ◯ No effect at all
- ◯ A small effect
- ◯ A large effect

12) Because of the difficulty in enrolling and monitoring patients, research conducted during pandemics is of lower quality than that conducted in the absence of a pandemic.

- ◯ Strongly disagree
- ◯ Somewhat disagree
- ◯ Neutral
- ◯ Somewhat agree
- ◯ Strongly agree

13) In the absence of a pandemic, do you believe that non-identifiable observational data from patients to inform clinical management should be collected with a waiver of consent?

Not at all — Neutral — Absolutely

(Place a mark on the scale above)

14) During a pandemic, do you believe that non-identifiable observational data from patients to inform clinical management should be collected with a waiver of consent?

Not at all — Neutral — Absolutely

(Place a mark on the scale above)

15) In the absence of a pandemic, do you feel comfortable randomizing your patients using deferred consent for an antiviral medication that has not been proven to be safe in your patient population?

- ◯ Yes
- ◯ No

16) During a pandemic, do you feel comfortable randomizing your patients using deferred consent for an antiviral medication that has not been proven to be safe in your patient population?

- ◯ Yes
- ◯ No

17) How would you feel about enrolling patients in an ethically approved study where patients are randomised to 1 of 2 currently used and acceptable treatments (e.g. steroid administration) and then followed up using routinely collected data, under a waiver of consent, in the absence of a pandemic?

- ◯ Very uncomfortable
- ◯ Somewhat uncomfortable
- ◯ Neutral
- ◯ Somewhat comfortable
- ◯ Very comfortable

18) How would you feel about enrolling patients in an ethically approved study where patients are randomised to 1 of 2 currently used and acceptable treatments (e.g. steroid administration) and then followed up using routinely collected data, under a waiver of consent, during a pandemic?

- ◯ Very uncomfortable
- ◯ Somewhat uncomfortable
- ◯ Neutral
- ◯ Somewhat comfortable
- ◯ Very comfortable

19) Do you consider yourself more of a clinician or a researcher?

Clinician ☐☐ Researcher

(Place a mark on the scale above)
