# Supplementary material for: Clinician-researcher’s perspectives on clinical research during the COVID19 pandemic
Source: PLoS One. 2020 Dec 9;15(12):e0243525. doi: 10.1371/journal.pone.0243525 (PMC7725301; doi:10.1371/journal.pone.0243525)
Supplement: S2 Appendix — (DOCX) [file pone.0243525.s002.docx]

| **Checklist Item** | | **Completion and Comments** |
| --- | --- | --- |
| **Design** | Describe survey design | Included in methods |
| **IRB (Institutional Review Board) approval and informed consent process** | IRB approval | Included in methods |
|  | Informed consent | Included in methods; implied consent, with additional information provided at outset of study (see S1 Appendix) |
|  | Data protection | No personal information collected; all data stored via REDCap |
| **Development and pre-testing** | Development and testing | Survey was rapidly adapted from an unpublished European study used in 2013; it was built and tested on REDCap by authors prior to dissemination |
| **Recruitment process and description of the sample having access to the questionnaire** | Open survey versus closed survey | Open to all those in networks to which it was distributed |
|  | Contact mode | Yes, was made on the internet, described in full in methods |
|  | Advertising the survey | Included in methods |
| **Survey administration** | Web/E-mail | Included in methods |
|  | Context | Included in methods |
|  | Mandatory/voluntary | Included in methods |
|  | Incentives | None offered |
|  | Time/Date | Included in methods |
|  | Randomization of items or questionnaires | Not randomized |
|  | Adaptive questioning Number of Items | No adaptive questions needed |
|  | Number of screens (pages) | One page due to short survey |
|  | Completeness check | Not included at time of submission, but discussed in results. Given voluntary nature of survey and non-mandatory questions, no “rather not say” option was provided, particularly since no identifying data was collected. |
|  | Review step | Survey few pages, therefore not necessary |
| **Response rates** | Unique site visitor | ​Response rates are impossible to calculate, for example, given the unknown denominator |
|  | View rate (Ratio of unique sur- vey visitors/unique site visitors) | ​Difficult to track via REDCap |
|  | Participation rate (Ratio of unique visitors who agreed to participate/unique first survey page visitors) | This is included in our results section |
|  | Completion rate (Ratio of users who finished the survey/users who agreed to participate) | This is included in our results section |
| **Preventing multiple entries from the same individual** | Cookies used | No cookies were used, as there was little incentive for multiple entries from the same individual (including no remuneration). |
|  | IP check | We did not check IP addresses, as there was little incentive for multiple entries from the same individual (including no remuneration). |
|  | Log file analysis Registration | None used. |
| **Analysis** | Handling of incomplete questionnaires | Questionnaires terminated early were included for the questions that were answered; for multi-question analyses, only those who had answered both questions were included. |
|  | Questionnaires submitted with an atypical timestamp | No timeframe cut-off was used. |
|  | Statistical correction | No weighting was used. |
